# A Multifaceted Computational Approach to Identify PAD4 Inhibitors for the Treatment of Rheumatoid Arthritis (RA)

**DOI:** 10.3390/metabo15030156

**Published:** 2025-02-25

**Authors:** Mansour S. Alturki, Mohamed S. Gomaa, Nada Tawfeeq, Abdulaziz H. Al Khzem, Mohsina B. Shaik, Murtadha Alshaikh Jafar, Mohammad Alsamen, Hasan Al Nahab, Mohammad Al-Eid, Alhassan Almutawah, Thankhoe A. Rants’o, Khaled A. G. Ayil, Mohammed Almaghrabi

**Affiliations:** 1 Department of Pharmaceutical Chemistry, College of Clinical Pharmacy, Imam Abdulrahman Bin Faisal University, Dammam 31441, Saudi Arabia; nztawfeeq@iau.edu.sa (N.T.); ahalkhzem@iau.edu.sa (A.H.A.K.); mbshaik@iau.edu.sa (M.B.S.); 2 College of Clinical Pharmacy, Imam Abdulrahman Bin Faisal University, Dammam 31441, Saudi Arabia; 2190001637@iau.edu.sa (M.A.J.); 2190001667@iau.edu.sa (M.A.); 2190001758@iau.edu.sa (H.A.N.); 2190001992@iau.edu.sa (M.A.-E.); 2190004681@iau.edu.sa (A.A.); 3 Department of Pharmacology and Toxicology, College of Pharmacy, University of Utah, Salt Lake City, UT 84112, USA; thankhoe.rantso@pharm.utah.edu; 4 Chemistry Department, Faculty of Science, King Abdulaziz University, Jeddah 21589, Saudi Arabia; kaliabdullah@stu.kau.edu.sa; 5 Department of Chemistry, Faculty of Science, Umm Al-Qura University, Makkah 21955, Saudi Arabia; 6 Pharmacognosy and Pharmaceutical Chemistry Department, Faculty of Pharmacy, Taibah University, Al Madinah Al Munawarah 30001, Saudi Arabia; mhmaghrabi@taibahu.edu.sa

**Keywords:** PAD4, PAD IV, rheumatoid arthritis, PAD4 activity, PAD4 inhibitor, citrullination, peptidyl arginine deiminase

## Abstract

Background/Objectives: Neutrophil cells’ lysis forms the extracellular traps (NETs) to counter the foreign body during insults to the body. Peptidyl arginine deiminase (PAD) participates in this process and is then released into the extracellular fluid with the lysed cell components. In some diseases, patients with abnormal function of PADs, especially PAD 4, tend to form autoantibodies against the abnormal citrullinated proteins that are the result of PAD activity on arginine side chains. Those antibodies, which are highly distinct in RA, are distinctly anti-citrullinated protein antibodies (ACPA). This study used an in-silico drug repurposing approach of FDA-approved medications to identify potential alternative medications that can inhibit this process and address solutions to the current limitations of existing therapies. Methods: We utilized Maestro Schrödinger as a computational tool for preparing and docking simulations on the PAD 4 enzyme crystal structure that is retrieved from RCSB Protein Data Bank (PDB ID: 4X8G) while the docked FDA-approved medications are obtained from the Zinc 15 database. The protein was bound to GSK 199—an investigational compound—as a positive control for the docked molecules. Preparation of the protein was performed by Schrödinger Protein Preparation Wizard tool. Binding pocket determination was performed by Glide software (Schrödinger Release 2021–3:Schrödinger, LLC., New York, NY, USA, 2021). and validation of molecular docking was carried out through the redocking of GSK 199 and superimposition. After that, standard and induced fit docking were performed. Results/Conclusions: Among the four obtained hits Pemetrexed, Leucovorin, Chlordiazepoxide, and Ioversol, which showed the highest XP scores providing favorable binding interactions. The induced-fit docking (IFD) results displayed the strong binding affinities of Ioversol, Pemetrexed, Leucovorin, Chlordiazepoxide in the order IFD values −11.617, −10.599, −10.521, −9.988, respectively. This research investigates Pemetrexed, Leucovorin, Chlordiazepoxide, and Ioversol as potential repurposing agents in the treatment of rheumatoid arthritis (RA) as they are identified as PAD4 inhibitors.

## 1. Introduction

**Figure 1:**
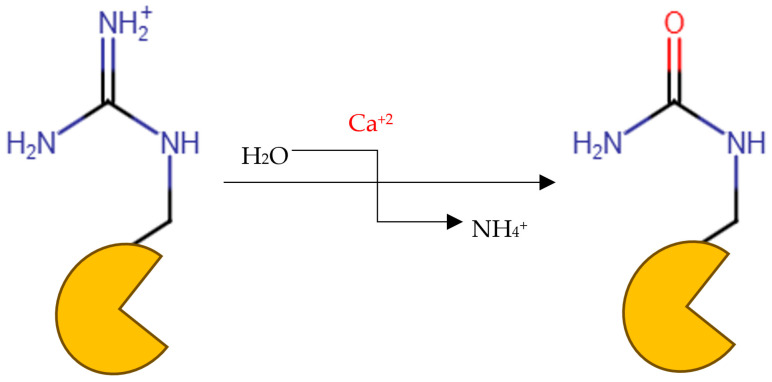
Protein citrullination reaction.

Peptidyl arginine deiminase 4 (PAD4) is an enzyme that catalyzes the conversion of peptidyl-arginine to peptidyl-citrulline in what is called citrullination (Figure 1) [1,2,3]. The enzyme plays a critical role in the regulation of numerous cellular functions, which include gene expression, programmed cell death, immune response, and cell division, as well as the construction of neutrophil extracellular traps (NETs) germane to the immune reaction against infections [4,5]. However, the dysregulation of this enzyme has been associated with the pathogenesis of many autoimmune and inflammatory diseases, such as rheumatoid arthritis (RA), cardiovascular disorders, multiple sclerosis, and neurodegenerative diseases such as Alzheimer’s and Parkinson’s diseases [5,6]. Histones serve as the structural framework for DNA in eukaryotes. Histones undergo many post-translational modifications, such as citrullination, phosphorylation, methylation, acetylation, and ubiquitination [7,8,9], and PAD enzymes play a part in these modifications.

Five isozymes are found in the human PAD family: PADs 1, 2, 3, 4, and 6 (calcium-dependent), showing around 50% sequence similarities [2,10]. They exert different cellular functions and distribution in situ. The principal expression sites for PAD1 are the epidermis and uterus, while the PAD2 is found in skeletal muscle, brain, inflammatory cells, cancer cell lines, and secretory glands. PAD3 is primarily active in hair follicles and keratinocytes; however, PAD4 is crucial to granulocyte function, especially in neutrophilic extracellular trap (NET) formation and some types of cancer. PAD6 is mainly activated during oocyte and embryonic development [1,2,6].

While background expression of all PAD isozymes is intracellular, PAD4 is the sole isozyme involved in the deimination of histones to histone citrullination. Most of the studies suggest that PAD2 could also take part in histone deimination, under certain conditions. Nonetheless, PAD4 and PAD2 are localized in cytoplasm and found in mitochondria and nucleus, thus broadening their biological roles. While histones seem to be the primary targets for PAD enzymes, their substrates include other proteins, such as fibrinogen, filaggrin, and actin. Citrullination of these proteins has a high risk of contributing to the pathogenesis of Rheumatoid Arthritis (RA), as abnormal citrullinated proteins are now recognized as antigens against which programmed autoreactive immune responses are mounted [1,2,11,12].

In the event of inflammatory stimuli or immune attack, lysis of the neutrophils takes place with the release of intracellular proteins, which are central to NET formation for trapping and inactivating foreign bodies [13,14]. Retrieval of that critical information reveals that PAD4 supports the formation of such NETs and that the activation of PAD4 to release citrullinated proteins into the extracellular fluid works partly to instigate inflammation, noticed in RA and other disorders [15].

Patients with abnormal function of PADs tend to form autoantibodies against the abnormal citrullinated proteins. Anti-PAD4 antibodies, which are highly distinct in RA and linked to the presence of anti-citrullinated protein antibodies (ACPA) [16], are detected in RA patients’ serum. The isoforms PAD2 and PAD4 are most highly associated with rheumatoid arthritis (RA) and autoimmune diseases. PAD4 is essential in the etiology of cardiovascular disorders, autoimmunity, multiple sclerosis, lupus, Parkinson’s disease, malignancies, Alzheimer’s disease, and more. This gives us a target to consider in various diseases’ management [1,2,3,17]. Anti-PAD4 antibodies can suppress or augment the enzymes’ activity, reducing or enhancing the inflammatory burden [18,19] and destroying nearby organs—most reports of Anti-PAD4 antibodies are in RA.

In the search for small-molecule PAD4 inhibitors, a series of different types have been considered, such as inhibitors like GSK199 and GSK484 and amidine derivatives (e.g., Cl-amidine), which show excellent in vitro efficacy. For example, amidine-based inhibitors exhibit high potency but are limited by their toxicity profiles. Additionally, the clinical application of current PAD4 inhibitors has been hampered by issues related to bioavailability and off-target effects (Table 1) [2,20,21,22]. GSK compounds bind to calcium-free enzymes with a higher potency than the amidines with additional selectivity to PAD 4 [22,23,24,25,26].

Multiple studies on several inhibitors faced different problems regarding safety and pharmacokinetics, while the inhibitory activity is retained in vitro and in vivo.

**Figure 2:**
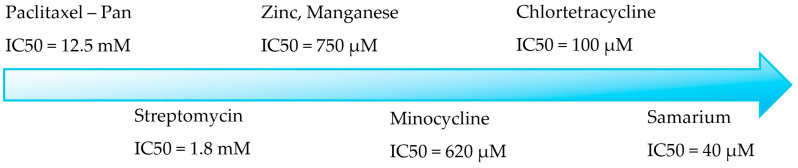
Known drugs with PAD 4 activity.

A few repurposed medications have a little inhibitory activity on PAD 4, like Streptomycin, chlortetracycline, minocycline, and Paclitaxel, while the investigational chloramines are more potent than them (Figure 2) [27,28,29].

This study employed two key paradigms in drug design: structure-based and ligand-based drug design. Molecular docking was employed to identify the top hits, which were then further validated by induced-fit docking, molecular mechanics with generalized Born and surface area solvation (MM-GBSA), and discrete Fourier transform (DFT) for verifying the binding efficiency. Structural similarity was assessed through shape-based screening. This study has been promising for improving efficacy in treatments directed at PAD4.

## 2. Materials and Methods

### 2.1. Computational Tools

Computational tools: Maestro 13.6 (Schrödinger 2023-2 version) was used to carry out computational simulations [30,31].

### 2.2. Generation of Databases and Ligand Library Preparation

**Figure 3:**
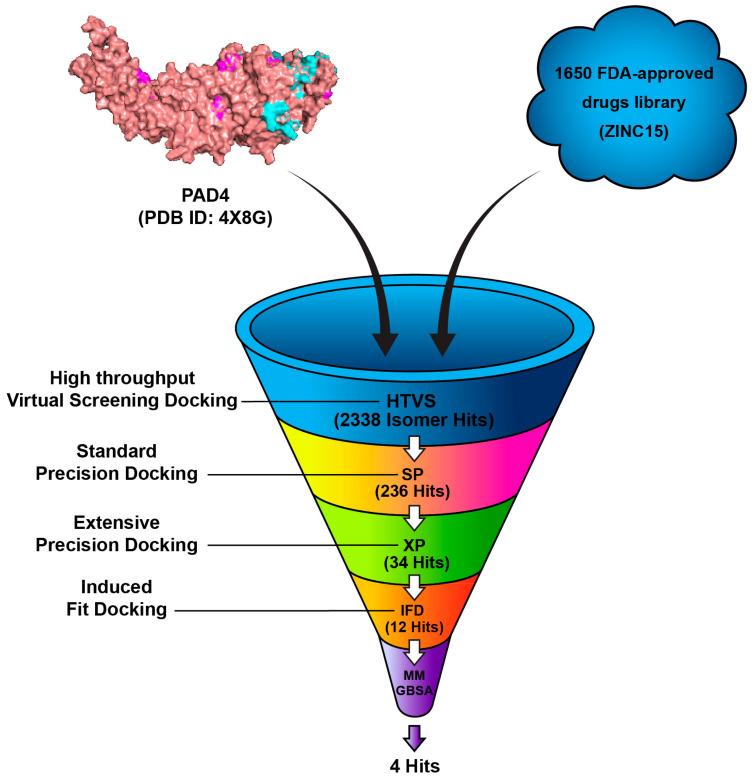
Workflow of the virtual screening process for identifying potential PAD4 inhibitors.

The FDA-approved drugs library was retrieved from the ZINC 15 online server (https://zinc.docking.org/ (accessed on 30 November 2022), a public web-accessible database containing over 750 million purchasable compounds [32]. A total of 1650 compounds were downloaded and saved in the 2D Structure-Data File (SDF) format and imported into Maestro. The chirality and ionization states were optimized at a physiological pH of 7.4 ± 2.0 and were determined with the aid of Epik. During this stage, several treatments were applied to the structures. Finally, the OPLS3 force field was selected to optimize the geometries [33]. The 1650 FDA-approved compounds were subjected to high-throughput virtual screening (HTVS) using Glide software to assess the binding potential to the target protein, PAD4 (PDB ID: 4X8G). A total of 2338 isomer hits were obtained, which were further filtered by Standard Precision (SP) docking. In this more selective docking procedure, 236 compounds were found to have favorable docking scores and plausible binding poses. These 236 compounds were docked by Extra Precision (XP) docking, which offered a highly accurate assessment of the ligand–receptor interactions and resulted in 34 compounds being identified. The 34 top compounds underwent induced-fit docking (IFD) and were further refined by MM-GBSA calculations predictive of binding affinity through estimating the free binding energy of the ligand–protein complexes. The final selection comprised of four compounds with the lowest estimated binding free energies and stable binding conformations, which are considered the best candidates for further investigation, as shown in Figure 3.

### 2.3. Crystal Structure Retrieval and Preparation

**Figure 4:**
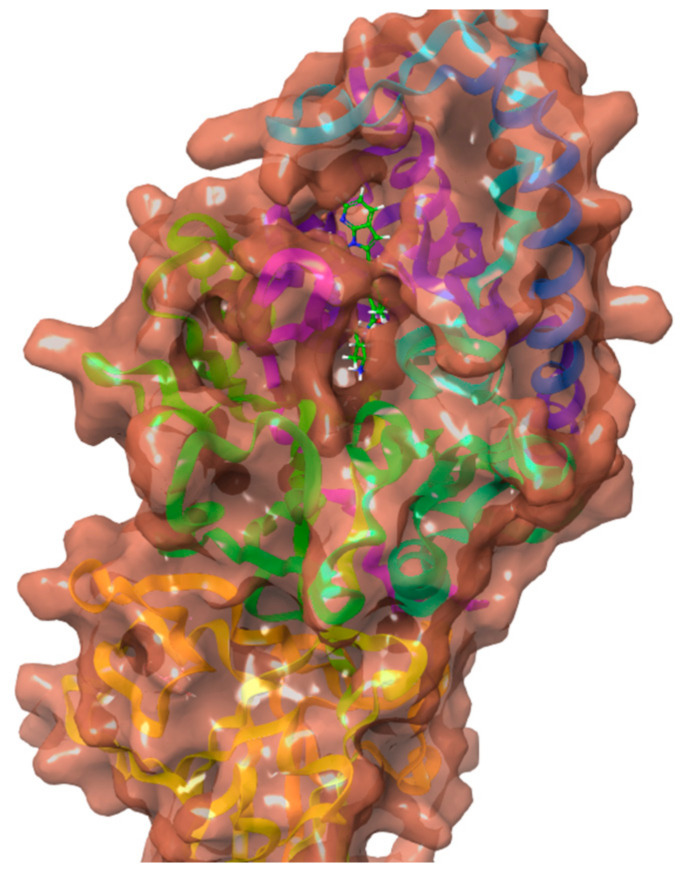
Crystal structure of Peptidyl arginine deaminase IV (PAD 4) bound to GSK199 (4X8G).

The structure of Peptidyl arginine deiminase IV bound to GSK199 was determined using X-ray crystallography and retrieved from the RCSB Protein Data Bank (PDB ID: 4X8G) at a resolution of 3.29 Å as displayed in Figure 4 [24]. The crystal structure was employed based on their previous use in similar studies and the relevance of the PAD4 inhibitors’ benchmark. The crystal structure was prepared using the Schrödinger Protein Preparation Wizard tool. Adjusting ionization at pH 7.4, adding missing amino acid residues and hydrogens, and removing extra water molecules. The protein structure was minimized and optimized by OPLS3 force field to enhance protein energies and avoid steric hindrances, with a default root mean square deviation (RMSD) value of 0.30 Å for non-hydrogen atoms.

### 2.4. Binding Pocket Determination and Validation of Molecular Docking

In Schrödinger’s Maestro, the binding pocket was identified using the workspace co-crystallized with PAD 4 and GSK 199. The Receptor grid generation tool was then utilized to create a docking grid by selecting the ligand-binding pocket from this crystal structure. A docking grid was established using Glide software, centered on the ligand-binding pocket from the PAD4 co-crystal structure, without restriction to evaluate potential off-target interactions and confirm the reliability of the identified binding site. For validation, the co-crystallized ligand was re-docked again in the same pocket. Superimposition in Maestro and RMSD calculations were used to confirm docking poses and interactions. Receptor grids were generated with a van der Waals radius scaling factor of 1.00 and a partial charge cutoff of 0.25. The docking procedure was subsequently repeated and verified using High-Throughput Virtual Screening (HTVS), Standard-Precision (SP) and Extra-Precision (XP) screening settings [34,35,36].

### 2.5. Standard Molecular Docking (Rigid)

The ligand was docked using the Glide tool without constraints, employing a vdw radius scaling factor of 0.80 and a partial charge cut-off of 0.15. The ligands’ flexibility was considered while the protein was considered a rigid structure, with all other parameters set to their default values. GlideScore was utilized to predict ligands’ binding affinity. The Pose Rank was utilized to identify the optimal docking pose for each ligand. Binding scores and conformation poses were used in detailed analysis of the resultant structures [31].

### 2.6. Induced-Fit Docking (IFD) (Flexible)

The IFD technique, developed by Schrödinger, is used to model ligands’ binding to different conformational changes. Each ligand undergoes initial docking using a softened potential (van der Waals radii scaling) and flexible conformational sampling. Side-chain prediction is conducted within specified distance of each ligand. Favorable binding poses of structures are predicted based on the IFD score [37].

### 2.7. Molecular Mechanics-Based Re-Scoring

Molecular mechanics generalized Born surface area (MM/GBSA) docking was used to improve the accuracy of affinity predictions of complexes [38,39].

ΔG binding free energy = ΔG binding, vacuum + ΔG solvation, complex −(ΔG solvation, ligand + ΔG solvation, receptor).

MM/GBSA allow both ligand and receptor flexibility, increasing the accuracy and ability to relate to normal physiology [40]. An intensive MM/GBSA simulation was used to rank the binding affinities of the five identified hits against the PAD 4 active site. Flexibility was incorporated by adjusting the distance between the hits or GSK 199 and PAD IV to 5 Å. The simulation employed the VGSB solvation model alongside the OPLS3 force field [41].

### 2.8. Shape-Based Screen

GSK 199 served as the reference structure on Schrödinger’s Shape Screening tool. Five compounds underwent screening utilizing the pharmacophore volume scoring technique, which evaluates each compound as an assembly of pharmacophore features, including aromatic groups, hydrogen bond acceptors (HBA), hydrogen bond donors (HBD), hydrophobic regions, as well as positively and negatively charged groups. The shape similarity score was taken from the highest number of matching features [42].

### 2.9. Quantum Chemical Calculations

The structural geometries of the four drugs selected with GSK199 for this study were optimized using quantum mechanics density functional theory (DFT) in the ground state with the hybrid functional method B3LYP [43,44], and the LanL2DZ basis set was applied [45]. The Gaussian 09 package was performed to optimize all the structures in the gas phase [42]. The output files of the optimized three-dimensional drugs were visualized using the GaussView 6.0 program [46]. The influential global reactivity descriptors of the chemical structures are determined based on the frontier molecular orbital theory (FMO) consisting of the highest occupied molecular orbital (HOMO) and the lowest unoccupied molecular orbital (LUMO), as given in Equations (1)–(7) [47,48,49,50,51,52,53]. In addition, the molecular electrostatic potential (MEP) and Mulliken population analysis are scrutinized to investigate the charge distributions and reactivity behavior of local atoms in selected drugs at the B3LYP/LanL2DZ level of theory [54,55].

(1) $$\mathrm{Energy}\;\mathrm{gap}\;(\mathrm{Egap})=E_{LUMO}-E_{HOMO}$$

(2) $$\mathrm{Ionization}\;\mathrm{potential}\;(\mathrm{IP})=-E_{HOMO}$$

(3) $$\mathrm{Electron}\;\mathrm{affinity}\;(\mathrm{EA})=-E_{LUMO}$$

(4) $$\mathrm{Global}\;\mathrm{hardness}\;(\eta )=\frac{IP-EA}{2}$$

(5) $$\mathrm{Global}\;\mathrm{softness}\;(\sigma )=\frac{1}{\eta }$$

(6) $$\mathrm{Electronegativity}\;(c)=\frac{IP+EA}{2}$$

(7) $$\mathrm{Electrophilicity}\;\mathrm{index}\;(ώ)=\frac{\chi^{2}}{2\eta }$$

## 3. Results and Discussion

### 3.1. Docking Studies

**Figure 5:**
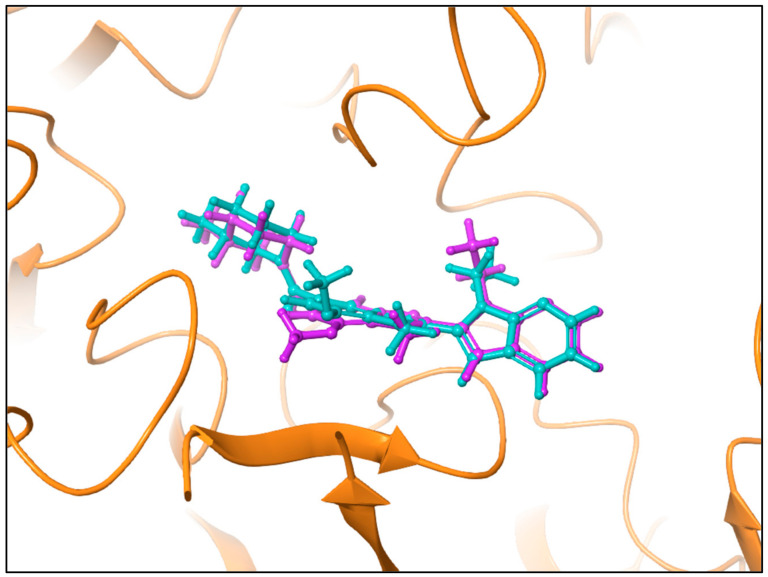
Comparison of binding poses of the co-crystallized ligand (Teal) and the redocked ligand (plum) within the PAD4 binding site, with an RMSD of 0.527.

**Figure 6:**
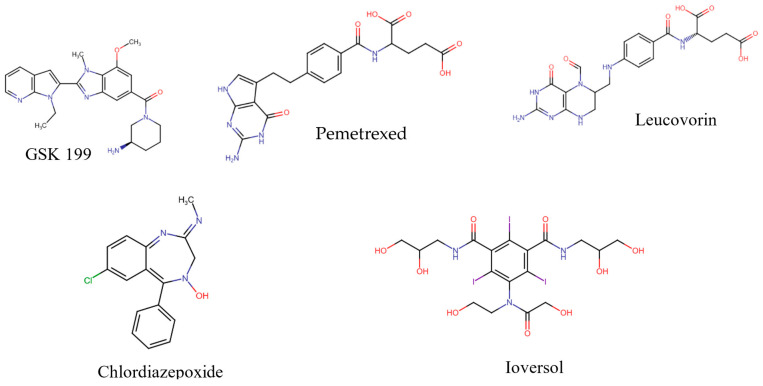
Potential inhibitors and positive standard (GSK199).

The co-crystalline ligand (GSK199) was first redocked into its target PAD4 using the same procedure and protocol applied for the FDA ligands to validate docking. Subsequently, rigid-body superposition was performed using Maestro’s structure superposition tool to align the predicted lowest energy conformation of the target with its corresponding co-crystalline ligand. The classical RMSD from the co-crystalline pose was calculated for the predicted binding poses, with an RMSD < 2 Å considered an effective threshold for validating correctly posed molecules [33,34]. The results showed good binding mode superimposition, with an RMSD of 0.527 for GSK199, reflecting the accuracy of Glide’s pose prediction Figure 5. The XP docking study identified four hits, including Pemetrexed, Leucovorin, Chlordiazepoxide, and Ioversol, as shown in Figure 6. The binding affinities of the four hits were assessed against PAD4. Initially, the inhibition profiles of these four hits were examined by docking them into the binding pockets of the target, investigating their binding patterns, target interactions, and binding affinities compared to the reference GSK199.

### 3.2. Computational Analysis of the Four Hits Binding to PAD4

**Figure 7:**
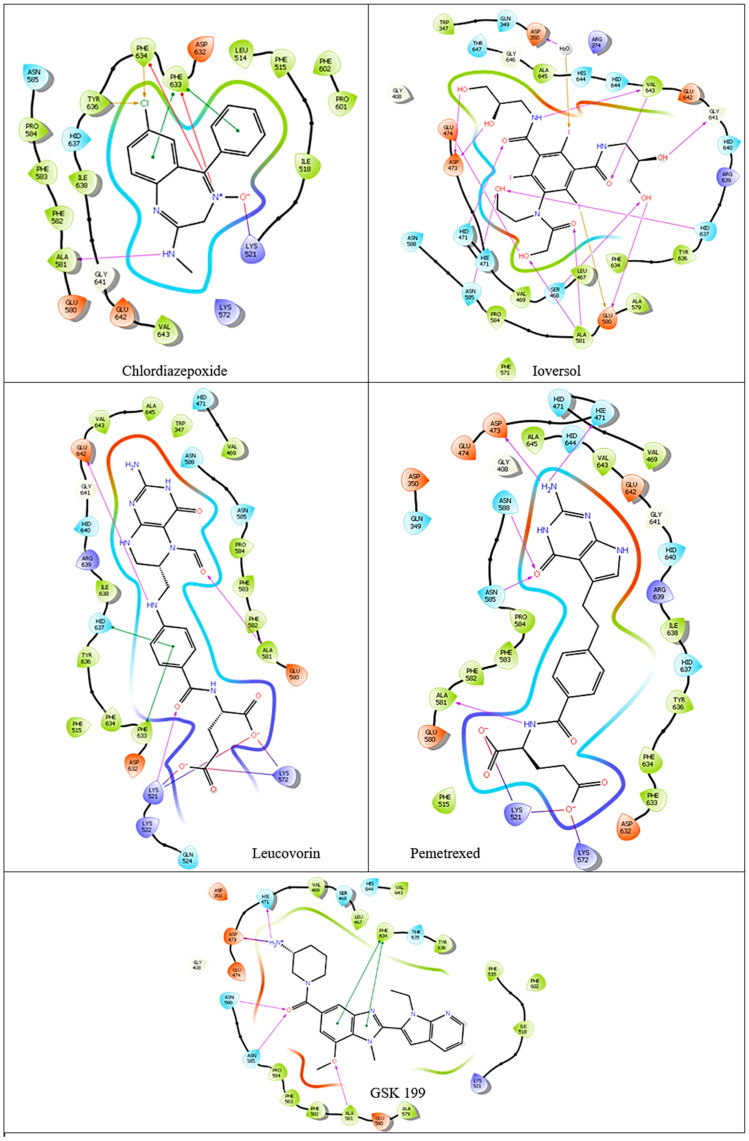
Two-dimensional ligand–protein binding interactions of PAD4 bounded to top four hit candidates and crystal ligand.

**Figure 8:**
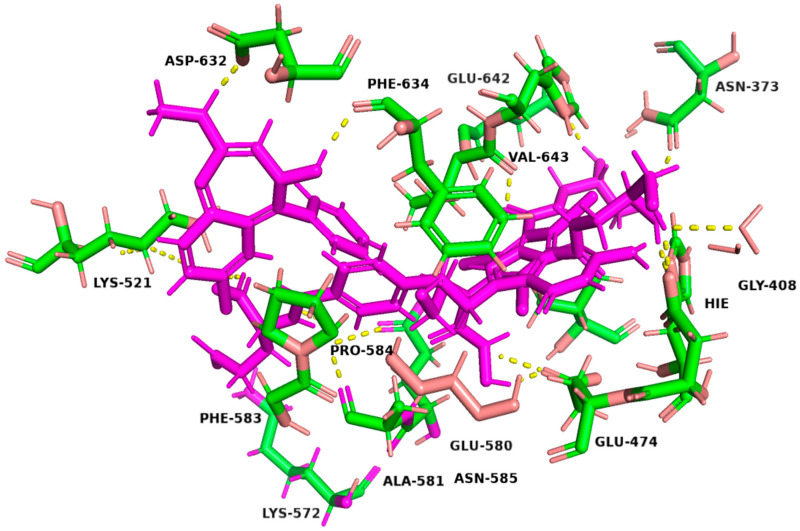
Three-dimensional representation of the binding interactions between the four hits and PAD4 binding pocket (PDB ID: 4X8G). Ligand atoms are shown as sticks (carbon atoms colored in magenta) and the key residues are shown as sticks (carbon atoms colored in green). Potential electrostatic interactions are represented as yellow dotted lines.

The binding affinities and interactions of the four hit compounds with Peptidyl Arginine Deiminase IV (PAD4) were evaluated using both rigid and induced-fit docking (IFD) methodologies (Table 2) [56]. The compounds investigated were Ioversol, Pemetrexed, Chlordiazepoxide, and Leucovorin, with GSK199 serving as a control. Ioversol emerged as the most promising hit, exhibiting superior docking scores and more favorable amino acid interactions than the other tested compounds. The IFD score for Ioversol was −11.617, indicating a robust binding affinity. The compound formed several critical hydrogen bonds with residues within the binding pocket of PAD4, including HID471, ASP473, GLU474, GLU580, ALA581, GLY641, and a conserved water molecule. These interactions are crucial for the stability and specificity of Ioversol within the PAD4 binding pocket. Pemetrexed also demonstrated a high IFD score of −10.599 and established interactions with key residues such as LYS521, LYS572, HIE471, ASP473, ASN585, ASN588, and ALA581. Chlordiazepoxide and Leucovorin displayed IFD scores of −9.988 and −10.521, respectively. Chlordiazepoxide interacted with residues LYS521, PHE633, PHE634, and ALA581, while Leucovorin formed bonds with LYS521, LYS572, LYS521, ALA581, GLU642, PHE633, and HID637. Favorable binding-based 2D and 3D docking positions interact with key residues within the binding pocket, as shown in Figure 7 and Figure 8.

### 3.3. Binding Free Energies Analysis

The MM-GBSA (Molecular Mechanics Generalized Born Surface Area) binding free energy analysis for the four compounds Ioversol, Leucovorin, Chlordiazepoxide, and Pemetrexed, along with the control GSK199, revealed significant differences in their binding affinities to PAD4 (Table 3) [57]. Ioversol has the most favorable net binding free energy of −53.53 kcal/mol, establishing better binding stability and affinity over all other compounds. Leucovorin exhibited a net binding free energy of −43.71 kcal/mol, which was notably less favorable than Ioversol. Chlordiazepoxide and Pemetrexed showed even lower binding affinities, with net binding free energies of −30.96 kcal/mol and −28.46 kcal/mol, respectively.

### 3.4. Shape Similarity Prediction

Table 4 displays the shape similarity scores of four compounds (Ioversol, Pemetrexed, Chlordiazepoxide, and Leucovorin) assessed against GSK199 as a reference [58]. The shape similarity analysis is crucial for understanding how well these compounds mimic the three-dimensional shape of the known inhibitor GSK199, which can influence their ability to bind to the target enzyme, Peptidyl Arginine Deiminase IV (PAD4). Pemetrexed demonstrated the highest shape similarity score of 0.300 among the compounds evaluated, indicating its structural resemblance to GSK199. Chlordiazepoxide and Leucovorin exhibited shape similarity scores of 0.280 and 0.258, respectively.

### 3.5. DFT Optimization Structures

**Figure 9:**
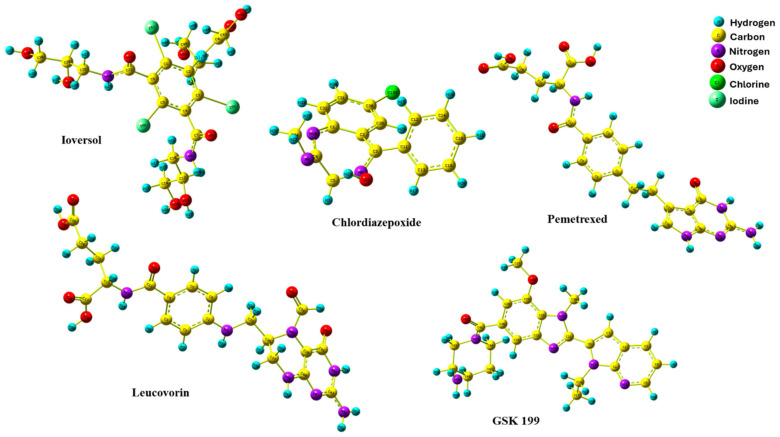
Optimized geometries of four promising drugs candidate with GSK 199.

The DFT method is a crucial quantum technique that provides valuable structure optimization details of electronic features relating to the minimum energy of molecules in three dimensions. This aspect of DFT empowers us with a deeper understanding of the conduct of organic compounds in biological systems, making it an essential tool in many previous studies [59,60,61,62]. Figure 9 shows the optimized geometries of the four hits and GK199. All compounds were obtained with no imaginary frequency to ensure the drugs were at minimal energy. In addition, the bond distances of all optimized drugs were determined in angstrom units using the B3LYP/LanL2DZ level of theory, as shown in Figure S1. Chemical bond distances of four potential inhibitors with GSK 199.

### 3.6. Frontier Molecular Orbital

**Figure 10:**
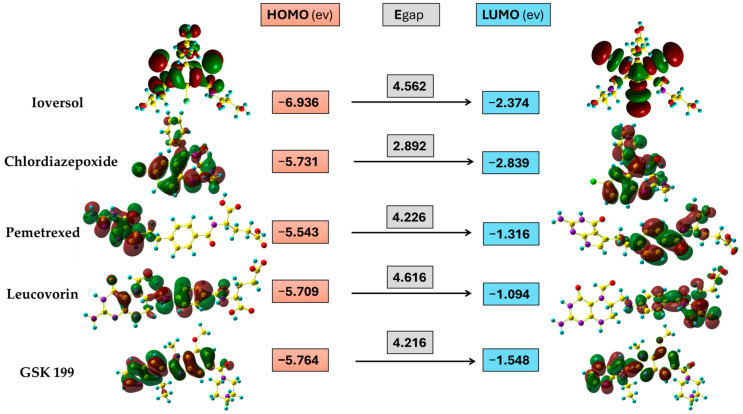
Three-dimensional representation of HOMO and LUMO distribution drugs, and the energy level of HOMO, LUMO, and energy gap (ev) values.

FMO is an essential tool obtained from the DFT quantum calculation method. It describes the energy of the highest orbital consisting of electrons (EHOMO) and the energy of the lowest empty orbital level (ELUMO). These energy levels determine a molecule’s reactivity and provide substantial evidence regarding its stability. As shown in Figure 10, the HOMO orbital of Ioversol is distributed at the whole molecule except the iodo derivative with side chains of terminals hydroxyl groups. In contrast, the LUMO is attributed to the triiodo phenyl derivatives with amide groups. In addition, the HOMO and LUMO of Chlordiazepoxide were lying around the whole drug except for the hydrogen of phenyl groups and LUMO of p-chloro phenyl derivative. Furthermore, the HOMO of Pemetrexed is mainly distributed on 2-amino-3,7-dihydro-4H-pyrrolo[2,3-d] pyrimidine-4-one as the donor group, while the LUMO orbital is found on the acceptor group of the carboxylic chain. Moreover, the HOMO orbital of Leucovorin is mainly located from the formyl piperazine derivative reaching central 4-aminobenzamide moiety. Meanwhile, the LUMO was almost like the LUMO of Pemetrexed. Additionally, the HOMO and LUMO orbitals of GSK199 were in the nearly identical region located from the center of the Benzimidazole scaffold to the pyrrolo[2,3-b] pyridine terminal.

### 3.7. Global Chemical Descriptors

The quantum chemical reactivity descriptors are a fundamental approach to scrutinizing the stability and reactivity of molecules according to Koopmans’ approximation [63]. The energy gap is a significant parameter that gives information about the stability of molecules. Overall, it is clear from Figure 10 that Chlordiazepoxide had the lowest Egap value (2.892 ev), indicating that it was the most kinetically unstable among the potential drugs, which might be due to the seven-membered ring. Meanwhile, the Egap values of four selected drugs were generally close together. The Egap tendency increases in the following order: Chlordiazepoxide < GSK199 < Pemetrexed < Ioversol < Leucovorin. On the other hand, global hardness and softness are vital criteria for estimating a compound’s reactivity and correlate to the Egap. A decline in hardness and a rising softness value indicates a lower energy gap and unstable compounds that are more likely to undergo a reaction. Table 5 shows that Chlordiazepoxide has the lowest hardness and the highest softness value. It was more reactive (unstable) compared to the other selected drugs. In contrast, Leucovorin was the most stable drug, containing a high hardness value and lower softness. At the same time, the GSK199, Pemetrexed, and Ioversol showed moderate reactivity compared to Chlordiazepoxide and Leucovorin. In addition, electronegativity is another helpful parameter that demonstrates the ability of atoms to attract electrons to itself in the chemical bonds in the molecule; the order of the calculated results is as follows, arranged in the growing trends of electronegativity values: Leucovorin < Pemetrexed < GSK199 < Chlordiazepoxide < Ioversol. Moreover, electrophilicity investigates compounds’ capability to accept electrons. The electrophilicity index order was diminishing in the rate from Leucovorin > Ioversol > Pemetrexed > GSK199 > Chlordiazepoxide. It was apparent that Ioversol, Pemetrexed, and GSK199 tend to accept electrons from the environment less than Leucovorin and more than Chlordiazepoxide.

### 3.8. Molecular Electrostatic Potential and Mulliken Population Analysis

**Figure 11:**
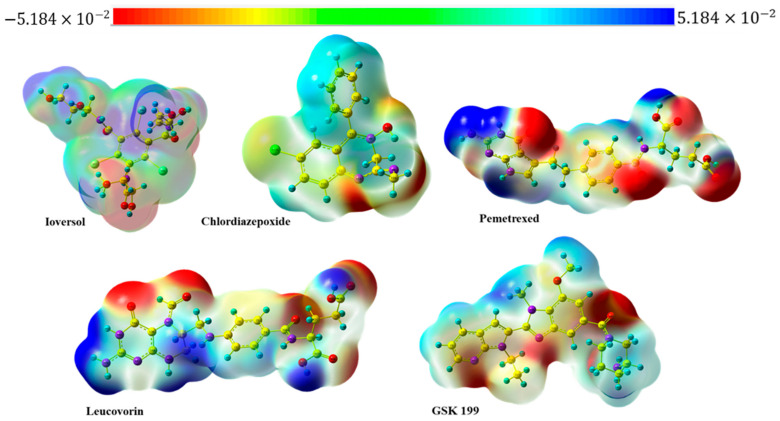
MEP map of four selected drugs and GSK 199.

The molecular electrostatic potential (MEP) is an essential tool for understanding the electron density of molecules around each atom. It is crucial to characterize which atom in a molecule tends to act as an electron donor or acceptor within biological systems. The MEP, as depicted in Figure 11, assists in identifying electron-rich and electron-deficient atoms. The red-to-orange hues represent the negative region, indicating potential electrophilic attack sites due to electron-rich atoms. Conversely, the positive area, indicated by blue-to-sky-blue cloud, is related to nucleophilic attack regions, representing electron-deficient atoms. Neutral atoms usually appear in white to green shades, which provides significant insights into the MEP mapping of selected drugs, the oxygen and nitrogen atoms emerging as the dominant regions of all drugs for electrophilic attack (electron-rich) atoms. At the same time, hydrogens attached directly to highly electronegative atoms are represented as a nucleophilic attack (electron-deficient) region. On the other hand, the Mulliken charge is another significant parameter that plays a crucial role in investigating the interaction of molecules with biological systems. It is beneficial in estimating the partial charge distribution at the atom level within a compound. A negative charge indicates an electron-dense atom with a potential region as a strong hydrogen bond acceptor (HBA). Conversely, the atom with positive values efficiently behaves as a hydrogen bond donor (HBD), as shown in Figure 11. The Ioversol, the oxygen of the hydroxyl group (O34), had the highest negative number (−0.537), indicating it had a significant role as an electron-rich atom. Meanwhile, the hydrogen (H35) attached to the same oxygen had the most positive value (0.381). The values of Mulliken atomic charges for the other selected drugs can be found in Figure S2. Mulliken charges of four optimized geometries drugs with GSK 199.

## 4. Conclusions

The current study presented an in silico drug repurposing technique of FDA-approved drugs for PAD 4 inhibition to identify potential alternative medications for RA. Four hits were identified including Pemetrexed, Leucovorin, Chlordiazepoxide, and Ioversol that showed the best XP scores together with favorable binding interactions. The IFD further supported the results and indicated a robust binding affinity with IFD scores of −11.617, −10.599, −10.521, and −9.988 for Ioversol, Pemetrexed, Leucovorin, and Chlordiazepoxide, respectively. The binding free energy calculation proved the favorable binding with scores ranging from −28.46 to −53.53 kcal/mol. The MM-GBSA calculation maintained Ioversol as the first hit while it repositioned Leucovorin, and Chlordiazepoxide as the second and third hits rather than the third and fourth, respectively. Furthermore, the binding potentials of the four hits and the crystal ligand were analyzed using DFT calculations to ascertain the predicted favorable binding. Of special interest is the identification of Chlordiazepoxide as a potential ligand based on its initial central analgesic activity and tolerability that would position it as a promising candidate for future testing and clinical development.

## Figures and Tables

**Table 1:** Previous investigational compounds’ potencies.

Irreversible amidines (Pan inhibitors)	IC50 on PAD4
F-amidine	1.9 µM
Cl-amidine	22 µM
Reversible compounds (Selective)	
GSK199	200 nM
GSK484	50 nM

**Table 2:** Rigid, induced-fit docking scores, and key amino acid residues interactions (PDB entry: 4X8G).

Compound	Glide Score Docking (Rigid)	Induced-Fit Docking (IFD) (Flexible)	Ionic Interactions	H-Bond Interactions	Pi Pi-Bond Interactions
Ioversol	−8.35	−11.617	-	HID471 ASP473 GLU474 GLU580 ALA581 GLY641 H_2_O350	-
Pemetrexed	−8.28	−10.599	LYS521 LYS572	HIE471 ASP473 ASN585 ASN588 ALA581	-
Chlordiazepoxide	−5.23	−9.988	LYS521 PHE633 PHE634	ALA581	PHE633
Leucovorin	−4.16	−10.521	LYS521 LYS572	LYS521 ALA581 GLU642	PHE633 HID637
GSK199	−9.58	-	HIE471 ASP473	ALA581 ASN585 ASN588	PHE634

**Table 3:** MM-GBSA net binding energy of the compounds/control.

Compound	*ΔG binding ^a^*
Ioversol	*−53.53*
Leucovorin	*−43.71*
Chlordiazepoxide	*−30.96*
Pemetrexed	*−28.46*
GSK199 (Control)	*−107.15*

^a^ Lower negative value indicates higher binding interactions within the binding pocket.

**Table 4:** Shape similarity of the hits and control. Similarity ranges: 0.5–1 (High), ≥0.3–0.49 (Intermediate), <0.3 (Low). Cutoff score ≥ 0.4.

Compound	Shape Similarity ^a^
Ioversol	0.212
Leucovorin	0.258
Chlordiazepoxide	0.280
Pemetrexed	0.300
GSK199	1

^a^ Values closer to 1 indicate higher shape similarity to GSK199.

**Table 5:** Quantum chemical reactivity parameters of top selected drugs with GSK 199.

*Compound*	HOMO	LUMO	Global Hardness (η)	Global Softness (σ)	Electronegativity (χ)	Electrophilicity Index (ώ)
*Ioversol*	−6.936	−2.374	2.281	0.438	4.655	5.934
*Pemetrexed*	−5.543	−1.316	2.113	0.473	3.430	4.718
*Chlordiazepoxide*	−5.731	−2.839	1.446	0.691	4.285	1.512
*Leucovorin*	−5.709	−1.094	2.308	0.433	3.402	6.147
*GSK199*	−5.764	−1.548	2.108	0.474	3.656	4.685

## Data Availability

All data generated or analyzed during this study are included in this published article.

## Supplementary Materials

The following supporting information can be downloaded at: https://www.mdpi.com/article/10.3390/metabo15030156/s1, Figure S1: Chemical bond distances of four potential inhibitors with GSK 199. Figure S2: Mulliken charges of four optimized geometries drugs with GSK 199.
