# Supplementary material for: A Multifaceted Computational Approach to Identify PAD4 Inhibitors for the Treatment of Rheumatoid Arthritis (RA)
Source: Metabolites. 2025 Feb 25;15(3):156. doi: 10.3390/metabo15030156 (PMC11943705; doi:10.3390/metabo15030156)
Supplement: Supplementary file 1 [file metabolites-15-00156-s001.zip › metabolites-3419314-supplementary.pdf]

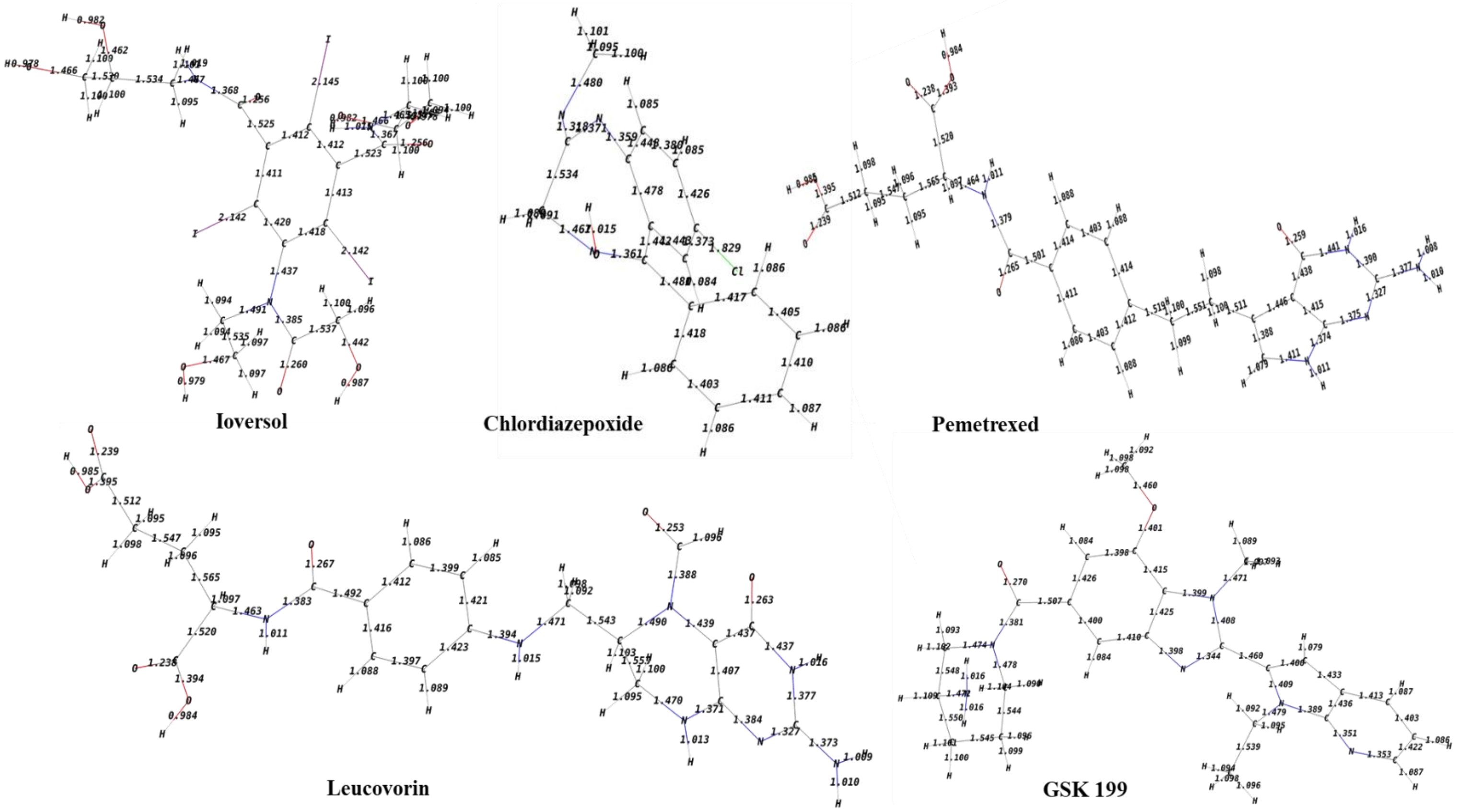

Figure S1. Chemical bond distances of four potential inhibitors with GSK 199.

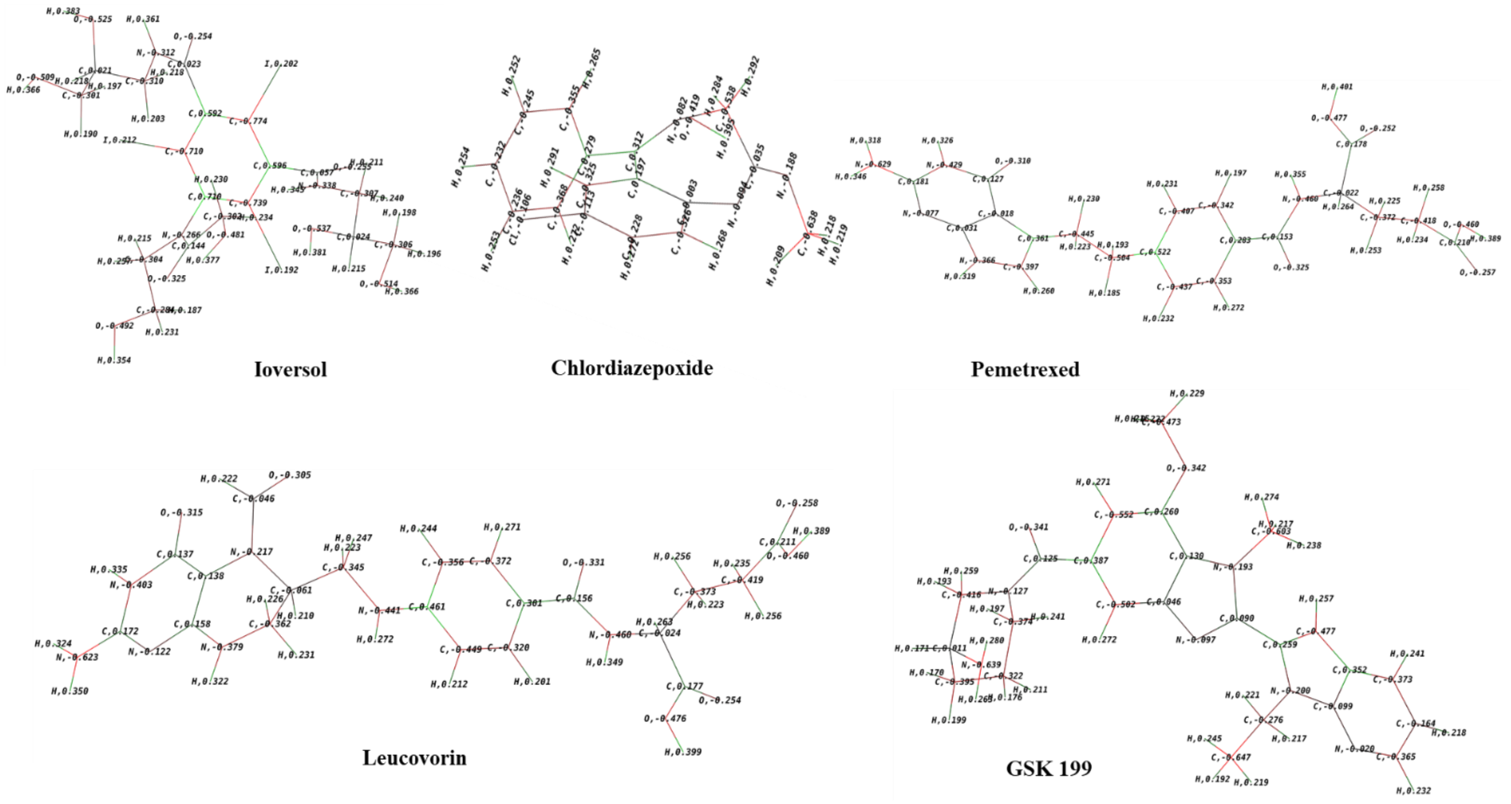

Figure S2. Mulliken charges of four optimized geometries drugs with GSK 199.
